# Comparative effectiveness of hybrid and laparoscopic techniques for repairing complex incisional ventral hernias: a systematic review and meta-analysis

**DOI:** 10.1186/s12893-023-02254-6

**Published:** 2023-11-16

**Authors:** Quan Wu, Weijie Ma, Qianqian Wang, Yaqi Liu, Yaokai Xu

**Affiliations:** 1 Department of General Surgery, Beijing Jishuitan Hospital, Capital Medical University, 31 Xinjiekou East Street, Xicheng District, Beijing, 100035 China; 2 Department of Pathology and Laboratory Medicine, Dartmouth Hitchcock Medical Center, Geisel School of Medicine at Dartmouth, Lebanon, NH USA; 3 Department of Epidemiology and Biostatistics, Beijing Research Institute of Traumatology and Orthopaedics, Beijing Jishuitan Hospital, Beijing, 100035 China

**Keywords:** Hybrid hernia repair technique, Laparoscopic hernia repair technique, Incisional ventral hernias, Meta-analysis

## Abstract

**Background:**

The recently developed Hybrid Hernia Repair technique (HHR), an adaptation of the laparoscopic method, has been proposed as a potential alternative for the treatment of complex Incisional Ventral Hernias (IVH). While single-arm studies have reported promising outcomes, a comprehensive meta-analysis affirming these benefits is lacking. This meta-analysis aims to compare the clinical outcomes of HHR and Laparoscopic Hernia Repair (LHR) in the management of IVH.

**Methods:**

An exhaustive search of the literature was conducted, targeting publications in both English and Chinese that compare HHR and LHR up to March 31, 2023. The primary outcomes examined were operation time, blood loss, and intestinal injury. Secondary outcomes included rates of seroma, wound infection, post-operative acute/chronic pain, recurrence, and mesh bulging. The RevMan 5.0 software facilitated the statistical meta-analysis.

**Results:**

The final analysis incorporated data from 14 studies, encompassing a total of 1158 patients, with 555 undergoing HHR and 603 treated with LHR. Follow-up data, ranging from 12 to 88 months, were available in 12 out of the 14 identified studies. The HHR method was associated with a significantly lower risk of seroma (OR = 0.29, *P* = 0.0004), but a higher risk of wound infection (OR = 2.10, *P* = 0.04). No significant differences were observed between the two techniques regarding operation time, blood loss, intestinal injury, intestinal obstruction, post-operative pain, mesh bulging, and recurrence.

**Conclusions:**

The HHR technique did not demonstrate a clear advantage over LHR in reducing surgical complications, apart from a lower incidence of postoperative seroma. Surgeons with substantial expertise may choose to avoid incidental conversion or intentional hybrid procedures. Further research is needed to clarify the optimal surgical approach for IVH.

**Supplementary Information:**

The online version contains supplementary material available at 10.1186/s12893-023-02254-6.

## Introduction

Incisional ventral hernias (IVH) are more likely to occur in elderly or obese individuals who have previously undergone abdominal surgery with suboptimal suturing or experienced wound infections. Hernia repair is the only solution to address the abdominal defect, and it can be performed using either an open or minimally invasive IVH repair, including laparoscopic and robotic assisted technique. The laparoscopic approach for hernia repair was first introduced by Le Blanc and Booth in 1993, and it has since gained popularity due to its ability to minimize large subcutaneous flaps, reduce the risk of wound infection, and prevent transfascial suture and mesh bulging, in comparison to the open method [1–3]. Nonetheless, the laparoscopic approach accounts for an average of 2.4% conversion rate [4], primarily due to extensive intestinal adhesions. Additionally, this method may predispose patients to postoperative seroma in cases with large orifices when the hernia sac is not excised, or the defect is not closed. The superiority of either technique regarding recurrence rate control remains debatable [5, 6]. Circa 2000, a combination of open and laparoscopic techniques was proposed to address the limitations associated with both methods in IVH repair [7]. This approach has been referred to as a hybrid technique, endoscopically assisted, or limited conversion technique; however, a consensus on its definition has not been reached. The procedure typically involves initial laparoscopic adhesiolysis, or an intention to perform open adhesiolysis followed by conversion to an open approach for sac excision, defect closure, and subsequent mesh placement and fixation under pneumoperitoneum via transfascial sutures and/or metal tacks [3, 8–12], and this procedure also can be achieved by hybrid robotic-assisted surgery introduced into clinical practice two decades ago, with posterior component separation technique for huge defects if necessary [10].

While a limited number of double-arm cohort studies [13–15] in the English literature have reported favorable outcomes with reduced postoperative morbidities, such as lower rates of bowel injury, hematoma, wound infection, and shorter hospital stays, no meta-analysis has comprehensively confirmed these advantages to date. Van den Dop [16] combined these variables as surgical site occurrences, highlighting the need to further investigate the potential benefits of the Hybrid Hernia Repair (HHR) technique. In this study, we aim to elucidate the clinical outcomes of HHR compared to the Laparoscopic Hernia Repair (LHR) technique for the management of Incisional Ventral Hernias (IVH).

## Material and methods

### Search strategy and data extraction

This study was designed in accordance with the Preferred Reporting Items for Systematic Reviews and Meta-Analyses (PRISMA) guidelines. We conducted a literature search in the following databases: PubMed, Medline, Embase, Web of Science, Cochrane Library, CNKI, and WANFANG. Search terms and MeSH terms included “incisional ventral hernia,” “hybrid technique,” “endoscopically assisted,” “limited conversion,” and “laparoscopy repair.” The publication timeframe was set between 1996 and 31 March, 2023. Additionally, reference lists of identified articles were utilized for supplementary retrieval. The search was limited to articles published in English and Chinese languages.

Following the removal of duplicates, two authors (WQ and MW) independently assessed the eligibility of the studies by reviewing abstracts and full texts. In cases where a consensus could not be reached for a particular study, the final decision was voted by author WQQ. Data pertaining to study characteristics, such as demographics, pertinent surgical details, surgical complications, and prognosis, were extracted by authors LY and XY and recorded in an Excel spreadsheet.

### Inclusion and exclusion criteria

Both randomized and non-randomized clinical trials comparing the hybrid repair technique for IVH to the laparoscopic technique were included, while single-arm studies were excluded. In the hybrid operation, mesh placement was limited to either IPOM or IPOM-plus styles, characterized by intraperitoneal mesh placement repair; as such, the Minimally Invasive Less Open Sublay Operation (MILOS) was excluded [17]. Studies meeting any of the following criteria were also excluded: parastomal hernia, absence of hernia size description, animal studies, letters, reports, and conference abstracts.

### Quality assessment

Authors MW and WQQ independently conducted quality assessments. Risk of bias was evaluated using two methods, including the Risk of Bias in Non-Randomized Studies of Interventions (ROBINS-I) [18] and the Cochrane risk of bias tool [19]. The former was used to assess the non-randomized studies, and the latter was to evaluate the randomized trials.

### Statistical analysis

Data were reported as means ± standard deviation (X ± SD) for continuous variables and as odds ratios (OR) or risk ratios (RR) for dichotomous variables. Meta-analysis was conducted using Review Manager Version 5.0 software (The Cochrane Collaboration, Oxford, UK). Weighted mean difference (WMD) and OR/RR were used to evaluate treatment effects with corresponding 95% confidence intervals (CI) for continuous and categorical variables, respectively. Heterogeneity analysis was assessed by the I^2^ value, with an I^2^ value > 50% or *P* < 0.1 considered significant; the fixed-effects or random-effects model was then applied as appropriate. Forest plots were used to display the outcomes of this meta-analyses.

## Results

**Fig. 1:**
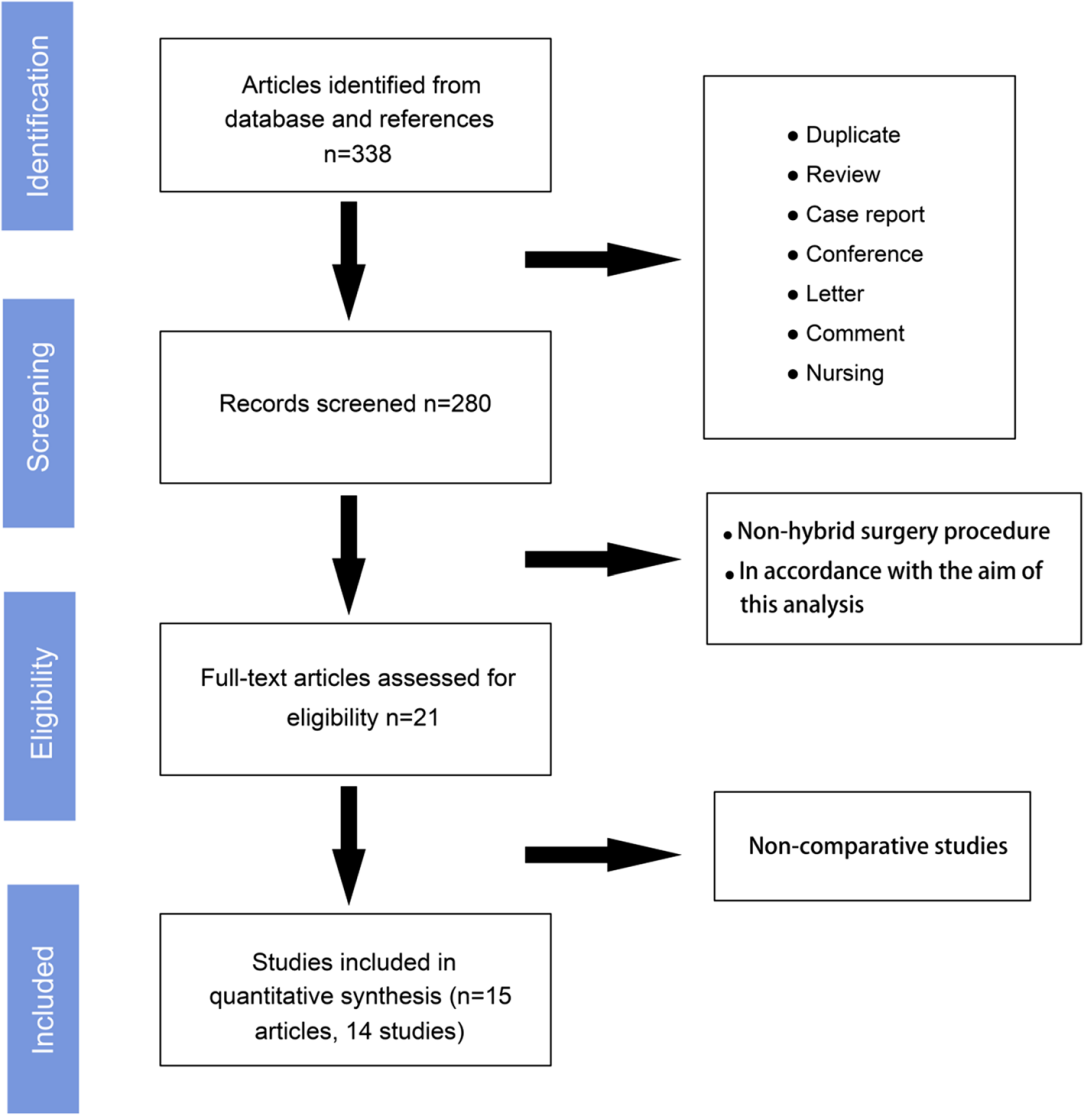
Flow Chart

The comprehensive search process is depicted in Fig. 1. After the elimination of duplicates, case reports, reviews, and articles not directly related to our objective, 21 articles addressing the hybrid technique were identified. Following a thorough full-text review, six non-comparative studies were excluded. Consequently, 15 relevant articles, comprising 14 studies with 555 cases in the HHR group and 603 in the LHR group, were included in the analysis [15, 20–33].

Among these, 13 were retrospective studies, and one was a randomized controlled trial (RCT), as detailed in Table 1. Two studies were assessed as low risk of bias, with the remaining exhibiting moderate risk (Table 1). The mean diameter of the hernia defects varied from 5.55 cm to 16.8 cm in the LHR group and from 6.34 cm to 21.50 cm in the HHR group (Table 2). Eight of the 14 studies documented the “classical” process of the hybrid technique. This process typically begins with laparoscopic exploration and adhesiolysis, followed by open surgery for hernia sac removal, patch placement within the abdominal cavity, and defect closure. The procedure concluded with the laparoscopic fixation of the patch. Three of the 14 studies replaced laparoscopic exploration and adhesiolysis with open surgery, with one suggesting that robotic operation could be a viable substitute for the laparoscopic phase.

**Table 1:** Baseline characteristics of included studies

Author	Year	Country	No. of patients	Design	Risk of bias
Deng [21]	2013	China		Retrospective	Moderate risk
*HHR*			20		
*LHR*			20		
Taqi [20]	2013	China		Retrospective	Moderate risk
*HHR*			5		
*LHR*			20		
Zhu [22]	2014	China		Retrospective	Moderate risk
*HHR*			102		
*LHR*			152		
Ozturk [23]	2015	Turkey		Retrospective	Moderate risk
*HHR*			16		
*LHR*			12		
Ye [24]	2015	China		Retrospective	Moderate risk
*HHR*			16		
*LHR*			20		
Wang [26]	2017	China		Retrospective	Moderate risk
*HHR*			25		
*LHR*			20		
Chen [27]	2017	China		Retrospective	Moderate risk
*HHR*			33		
*LHR*			37		
Ahonen [15]	2017	Finland		Retrospective	Moderate risk
*HHR*			24		
*LHR*			38		
Halka [25]	2018	United States		Retrospective	Low risk
*HHR*			25		
*LHR*			57		
Liu [29]	2019	China		Retrospective	Moderate risk
*HHR*			12		
*LHR*			23		
Zhao [30]	2019	China		Retrospective	Moderate risk
*HHR*			14		
*LHR*			16		
Ahonen [28, 31]	2018, 2020	Finland		RCT	Low risk
*HHR*			90		
*LHR*			94		
Tian [32]	2020	China		Retrospective	Moderate risk
*HHR*			20		
*LHR*			21		
Yang [33]	2022	China		Retrospective	Moderate risk
*HHR*			153		
*LHR*			73

**Table 2:** Patient and Surgical Characteristics

Author	Gender (F/M)	Age (year)	BMI (kg/m^2^)	Size of hernia defect (cm)	Recurrent hernia	Grade of adhesion	Process of hybrid method	Reduction of hernia sac	Closure of defects	Follow up (months)▲
Deng 2013						NA		Yes	Yes	63
*HHR*	10/10	58.9 ± 3.0	NA	16.07 ± 3.04	8/20		L-O-L^a^			
*LHR*	11/9	55.3 ± 3.3	NA	16.80 ± 2.16	6/20					
Taqi 2013						NA		Yes	Yes	21
*HHR*	3/2	65.2 ± 0.7	NA	9.6 ± 0.6	NA		L-O-L			
*LHR*	12/8	50.3 ± 2.5	NA	9.3 ± 0.2	NA					
Zhu 2014						NA		Yes	Yes	48
*HHR*	NA	NA	NA	> 10	NA		L-O-L			
*LHR*	NA	NA	NA	> 10	NA					
Ozturk 2015						NA		Yes	Yes	12
*HHR*	1/15	59	29.9	> 10	NA		O-L^b^			
*LHR*	3/9	58	30	> 10	NA					
Ye 2015						NA		Yes	Yes	48
*HHR*	10/6	55.9 ± 10.4	NA	11.75 ± 3.47	NA		O-L			
*LHR*	9/11	56.4 ± 10.0	NA	12.81 ± 4.17	NA					
Wang 2017						NA		NA	NA	60 (37.2^d^)
*HHR*	NA	65.2 ± 0.7	NA	13.60 ± 0.80	NA		NA			
*LHR*	NA	50.3 ± 2.5	NA	9.30 ± 0.20	NA					
Chen 2017						NA		Yes	Yes	30
*HHR*	NA	NA	NA	8.0 ± 6.0	NA		L-O-L			
*LHR*	NA	NA	NA	6.0 ± 4.0	NA					
Ahonen 2017						NA		NA	Yes	NA
*HHR*	16/8	58.0 ± 11.4	31.8 ± 5.6	6.34 ± 77.26	NA		NA			
*LHR*	24/14	61.0 ± 12.7	31 ± 6.3	5.55 ± 4.80	NA					
Halka 2017						NA				
*HHR*	9/16	61.5 ± 11.5	34.7 ± 6.8	21.50 ± 7.10	14/25		O-R^c^	Yes	Yes	NA
*LHR*	38/19	58.1 ± 13.9	33.6 ± 7.1	16.30 ± 5.80	23/57					
Liu 2019						NA		Yes	Yes	70 (26^d^)
*HHR*	8/4	NA	NA	12.38 ± 3.39	1/12		L-O-L			
*LHR*	22/1	NA	NA	6.67 ± 1.51	4/23					
Zhao 2019						NA		Yes	Yes	18
*HHR*	2/12	66.8 ± 10.6	NA	12.36 ± 4.15	NA		L-O-L			
*LHR*	2/14	65.6 ± 10.6	NA	11.48 ± 3.18	NA					
Ahonen 2018, 2020						NA		Yes	Yes	12
*HHR*	54/36	60.0 ± 12.8	NA	10.50 ± 8.90	NA		NA			
*LHR*	54/40	57 ± 11.4	NA	13.20 ± 11.10	NA					
Tian 2020						NA		Yes	Yes	12
*HHR*	13/7	66.6 ± 9.4	NA	8.95 ± 1.58	NA		L-O-L			
*LHR*	15/6	62.5 ± 7.4	NA	8.33 ± 1.83	NA					
Yang 2022						NA		Yes	Yes	88 (41^d^)
*HHR*	56/97	62.6 ± 15.5	NA	13.40 ± 3.40	NA		L-O-L			
*LHR*	27/46	60.8 ± 15.8	NA	12.70 ± 3.10	NA					

^a^ *L-O-L* represented the operation started with laparoscopy, then followed by open surgery and ended up with laparoscopy; ^b^ *O-L* represented the operation started with open surgery and ended up with laparoscopy; ^c^ *O-R* represented the operation started with open surgery and end up with robotic surgery. ▲ represented the maximum follow-up period and ^d^represented the median follow-up time. *NA* meant not available

### Primary outcomes: evaluations of surgical outcomes

**Fig. 2:**
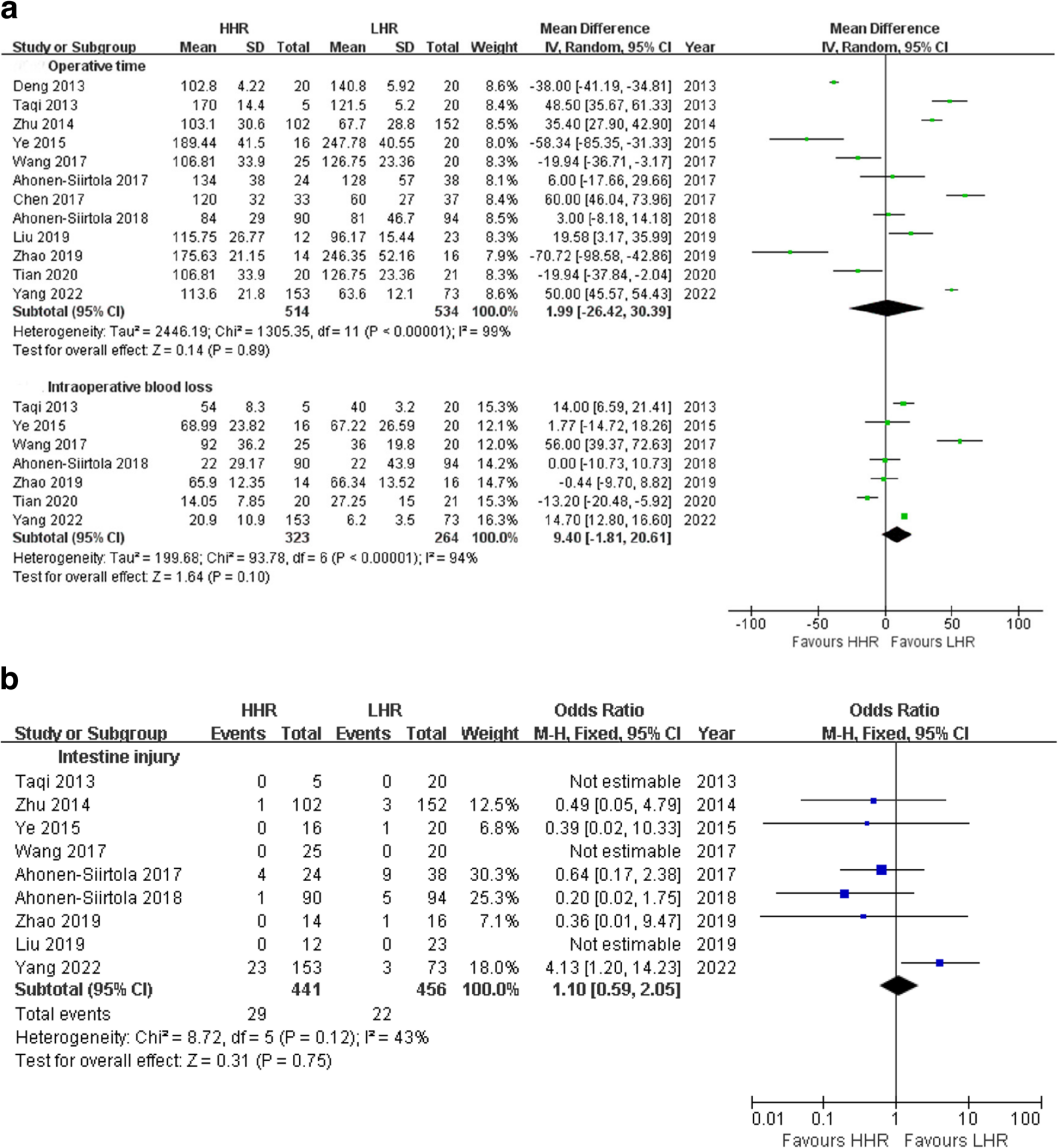
**a** Meta-analyses of primary outcomes: operative time and blood loss. **b** Meta-analyses of primary outcome: Intestine injury

Heterogeneity analysis for the comparison of operative time between HHR and LHR was based on 12 studies, as one study lacked standard deviation data [23]. The I^2^ value was 99%, with *p* < 0.000001. Therefore, the mean difference (MD = 1.99 min) was calculated using the random-effects model, indicating that the operative time for both methods was relatively similar (*p* = 0.89) (Fig. 2a). Intraoperative blood loss in HHR was slightly higher than that in LHR, with an MD of 9.40 ml, 95% CI [−1.81, 20.61], and *p* = 0.10, as determined from seven studies (Fig. 2a). Nine studies provided complete data on the incidence of intraoperative intestinal injury, with no heterogeneity observed (I^2^ = 40%, p = 0.14). The risk of intestinal injury did not demonstrate a significant preference between HHR and LHR (*p* = 0.75) (Fig. 2b).

### Secondary outcomes: assessment of postoperative morbidities

#### Seroma formation and wound infection

**Fig. 3:**
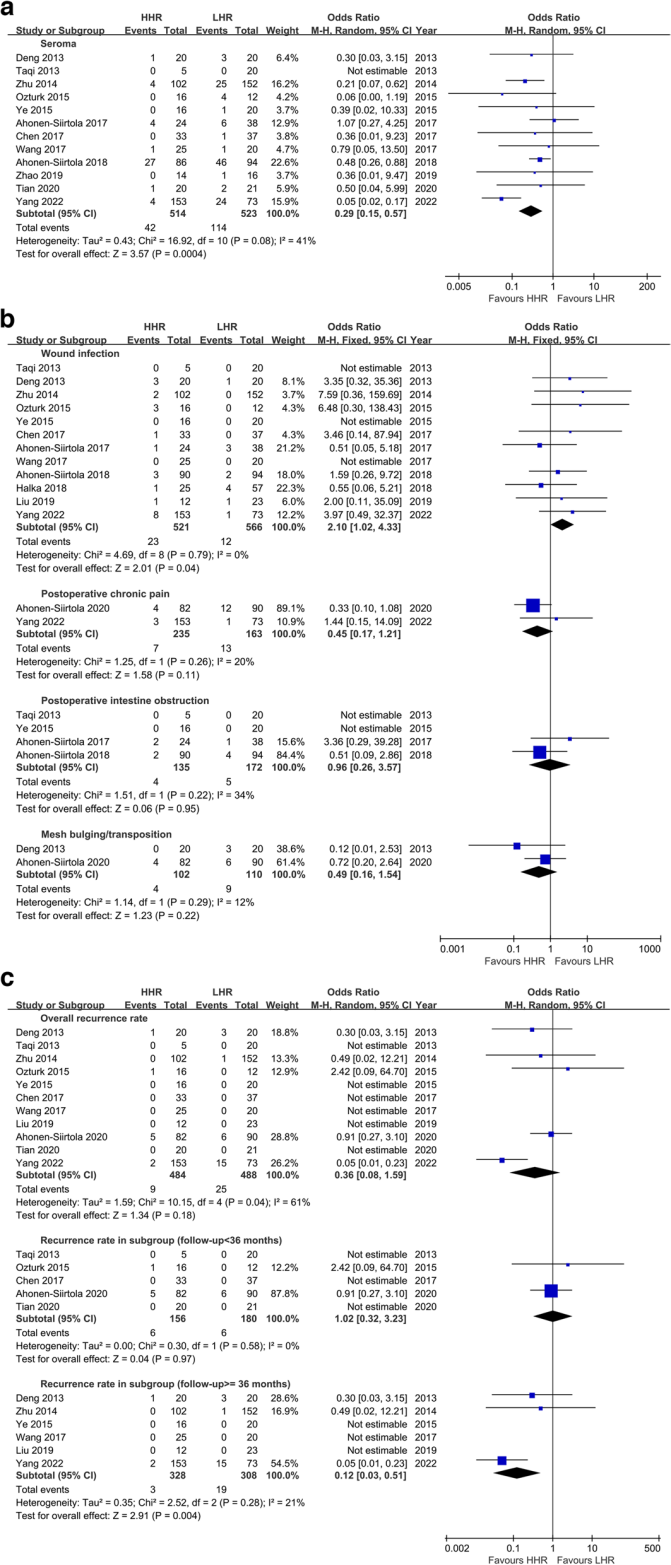
**a** Meta-analyses of secondary outcome: seroma formation. **b** Meta-analyses of dichotomous variables in the secondary outcomes. **c** Meta-analysis of recurrence rate and subgroup analysis

Subsequently, comparisons of short-term postoperative complications were performed, with a focus on seroma formation and wound infection. Twelve studies provided complete data for seroma formation analysis, revealing significant heterogeneity (I^2^ = 53%, *p* = 0.02) with the risk ratio (RR) effect measure. Upon correcting RR to odds ratio (OR) with the random-effects analysis model, heterogeneity decreased (I^2^ = 41%, *p* = 0.08). The results showed that the risk of seroma formation was significantly lower in HHR compared to LHR (OR = 0.29, 95% CI [0.15, 0.57], *p* = 0.0004) (Fig. 3a). A similar outcome (I^2^ = 33%, *P* = 0.14; OR = 0.25, *P* = 0.0006) was established when excluding one study [28, 31] with 1 month of seroma events different from the other included studies.

Interestingly, 12 out of 14 studies without heterogeneity (I^2^ = 0%, *p* = 0.79) demonstrated that the risk of wound infection in HHR was 2.1 times higher than in LHR (95% CI [1.02, 4.33], *p* = 0.04) (Fig. 3b).

#### Postoperative pain

**Fig. 4:**
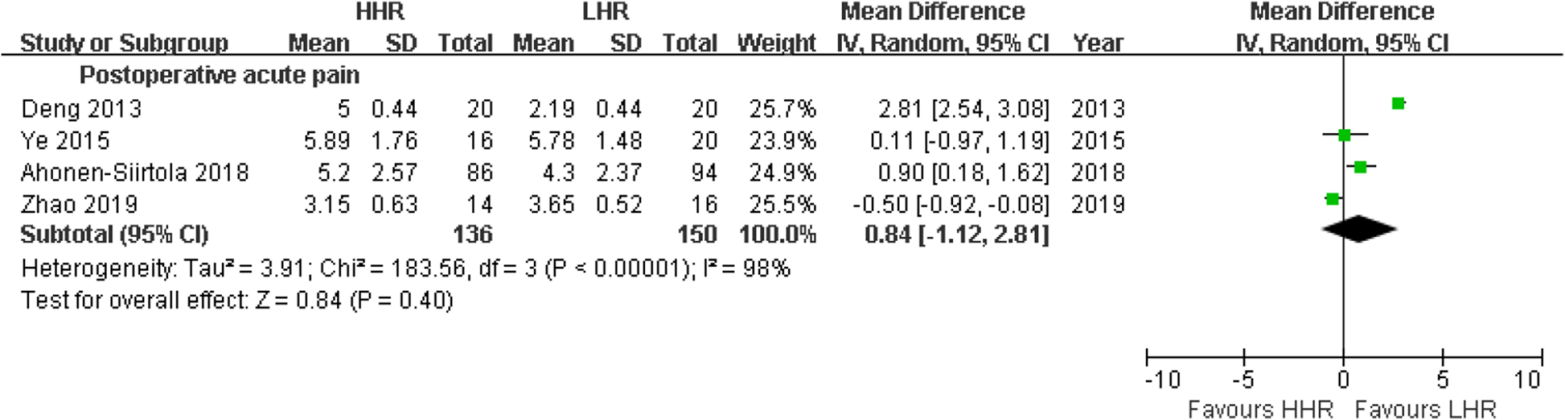
Meta-analyses of secondary outcome:postoperative acute pain

Additionally, patients in the HHR group experienced a similar extent of postoperative acute pain (VAS MD = 0.84 scores) compared to the LHR group in an analysis involving four studies with 136 vs. 150 cases (*p* = 0.40) (Fig. 4). Two studies with 235 vs. 163 cases assessing chronic pain showed a lower incidence rate in HHR compared to LHR, but the difference was not significant (Z = 1.58, *p* = 0.11) (Fig. 3b).

#### Postoperative intestine obstruction

Four studies analyzed the difference in intestinal obstruction incidence between the two methods, finding no statistically significant difference (OR = 0.96, 95% CI [0.26, 3.57], *p* = 0.95) (Fig. 3b).

#### Mesh bulging and recurrence

Twelve included studies had declared the maximum follow-up periods ranging from 12 to 88 months, with three [15, 25, 30] out of 14 studies that did not indicate the recurrent cases. None of the studies specified 1-year, 3-year, or 5-year data for further stratified analysis except one [31]. The OR value for overall recurrence rate between HHR and LHR was 0.36 (95% CI [0.08, 1.59], *p* = 0.18) with heterogeneity (I^2^ = 61%, *p* = 0.04), indicating no statistical difference in recurrence control between the two methods. Further subgroup analysis suggested that the comparison between the two methods in recurrence rates with follow-up less than 36 months did not achieve statistical difference (Z = 0.04, *P* = 0.97), while in another subgroup analysis with follow-up equal or greater than 36 months, the result indicated HHR had a lower risk of recurrence compared to LHR (OR = 0.12, 95% CI [0.03, 0.51], Z = 2.91, *P* = 0.004) (Fig. 3c).

Mesh bulging or transposition, which resembles hernia recurrence in appearance and causes patient dissatisfaction, was reported in only two studies, with no significant difference between HHR and LHR in bulging rates (OR = 0.49, 95% CI [0.16, 1.54], *P* = 0.22) (Fig. 3b).

## Discussion

The hybrid technique was proposed around 2000. Stoikes [3] once described its indications as follows: the anticipation of significant difficulty in adhesiolysis under a fully laparoscopic scenario, recurrent hernia with prior mesh, and avoidance of extensive subcutaneous flap when the hernia preferentially bulges toward one side of the abdomen. In such cases, the hybrid technique was considered a favorable option to decrease surgical complexity. However, unexpectedly, this meta-analysis demonstrated that HHR did not offer advantages in controlling operation time or blood loss compared to LHR. This finding is similar to previous meta-analyses comparing open and laparoscopic procedures, with neither achieving statistical significance, despite both indicating that the laparoscopic procedure took less surgical time than the open procedure (Lap vs. Open, SMD: − 1.83, *p* = 0.143 and SMD: −0.08, *p* = 0.97, respectively) [6, 34]. We speculate that this may be attributable not only to the more extensive surgical process in HHR compared to LHR, such as additional skin incisions, sac dissection, and closure of the orifice but also to complex morbidities or the extent of adhesion. Furthermore, the incidences of intestinal injury and obstruction between the two methods did not exhibit significant differences in 9 out of 14 studies, suggesting that experienced and proficient surgical performance can minimize potential risks occurring during dense adhesiolysis.

Cassar [35] summarized numerous studies prior to 2000, reporting that the rates of postoperative seroma formation in open procedures with mesh repair ranged from 1 to 15%, while in laparoscopic procedures, they ranged from 1 to 36%. However, a subsequent meta-analysis [34] suggested that the risk did not significantly favor patients undergoing laparoscopic repair compared to those undergoing open hernia repair (open vs. lap, OR = 1.54, *p* = 0.38). In contrast, the results of this meta-analysis supported the notion that HHR yielded a lower incidence of postoperative hematoma or seroma than LHR. Hernia sac excision and subcutaneous suction drainage have long been considered routine processes in hybrid hernia surgery, which may contribute to the reduced risk of postoperative seroma and hematoma formation [36].

In contrast to seroma and hematoma, which are often asymptomatic and predisposed to self-resolution, wound infection is of greater concern, as it can potentially lead to mesh infection and necessitate mesh removal. In this meta-analysis, the risk of wound infection was found to be more favorable for HHR than LHR, with HHR vs LHR yielding an OR of 2.10 (P = 0.04). This finding aligns with two earlier meta-analyses comparing open and laparoscopic surgeries [5, 34]. The higher risk of infection in open or combined open surgeries may be attributed to more extensive subcutaneous mobilization.

Postoperative pain is a common discomfort. In this analysis, four studies assessed pain within 1 week after surgery, referred to here as acute pain. No statistical significance was observed between the two groups in postoperative VAS scores. Sajid [6] compared the impact of acute pain between laparoscopic and open repair using two RCT trials. The results demonstrated that the laparoscopic approach did not show superiority, lap vs open, VAS SMD = − 0.04, *P* = 0.84, and similar outcomes were observed in two other later RCT trials [37, 38]. In contrast, chronic pain is defined as moderate or severe discomfort persisting for 6 to 8 weeks or even longer than 3 months after the procedure, as reported in various literatures [33, 39]. Chronic pain is relatively uncommon in the laparoscopic era. The incidence rate was reported as 1.6% (13/819) by Heniford [40] and 1.5% (6/389) by Franklin [41]. A recent systematic review [42] contributed a pooled incidence rate of 4.49% (0–15.3) for chronic pain in the HHR group, which is nearly as low as that in the LHR group. The causes of chronic pain are associated with skin incision, prosthetic material, and surgical technique, including tension-free procedures, mesh fixation, nerve injury, and nerve entrapment. Since the LHR and HHR methods are relatively similar in surgical procedures, they are expected to have no significant difference in the incidence rate of chronic pain. In this analysis, the risk preference did not show a significant discrepancy between the two techniques, despite the OR of 0.45 indicating the risk more prefer LHR, which appears to confirm the result mentioned above.

This analysis also demonstrated no significant variation in recurrence rates between HHR and LHR method. However, a pronounced heterogeneity was observed. To address this, we designated a minimum follow-up period of 36 months as a benchmark for evaluating hernia recurrence rates [4], establishing a cutoff point to facilitate subgroup analyses aimed at delineating the source of the heterogeneity. Notably, no heterogeneity was observed within the individual subgroups, indicating that the disparate follow-up durations across the selected trials may have contributed to the significant heterogeneity noted in the overall analysis. Due to the lack of specified data on 1-, 3-, or 5-year recurrence rates, it remains inconclusive whether the risk of recurrence beyond 36 months is more favorable with LHR compared to HHR based on the available trials featuring varied follow-up durations.

Hernia recurrence rates can vary based on the repair methods and materials used. Incisional hernias repaired by suturing have high recurrence rates (12–54%), while mesh repair is associated with recurrence rates ranging from 2 to 36% [6, 43]. Al Chalabi [5] summarized five randomized trials involving 611 IVH patients with follow-up periods ranging from 8 to 35 months, revealing a risk ratio for recurrence rate of 1.29 for laparoscopic versus open repair (95% CI [0.79, 2.11], *P* = 0.30). Awaiz [34] also reported a pooled OR of 1.41 for laparoscopic versus open repair (95% CI [0.81, 2.46], *P* = 0.23) based on six RCT trials consisting of 751 IVH patients with follow-up periods between 2 and 35 months. In the era of mesh repair, the likelihood of encountering high recurrence rates appears to be substantially reduced. Our findings reinforce the reliability of this conclusion, although there were few RCT trials included in this meta-analysis.

Mesh bulging, characterized by uneven protrusions in the area of previous hernia repair, is also referred to as pseudo-recurrence and has been associated with inadequate surgical techniques, particularly when employing the bridging technique without closure of defects [44, 45]. In this analysis, two included studies reported no significant differences in mesh bulging occurrence between the two methods. However, neither study specified whether the bridging technique was used in the laparoscopic procedures.

Several limitations are associated with this analysis. First, the inclusion of a large number of non-randomized and retrospective studies potentially increased the risk of selection bias. Second, methodological discrepancies existed among the included studies; for instance, some studies did not report whether defect closure and hernia sac dissection were performed. The HHR method was described as beginning with laparoscopy, transitioning to open, and concluding with laparoscopy; however, some studies reported laparoscopy followed by open, while others did not specify this aspect. Additionally, the evaluation of the extent of abdominal adhesions varied. Lastly, double-arm cohort studies comparing HHR with LHR are scarcely found in English literature. Interestingly, single-arm studies focusing solely on the effect of HHR are more prevalent. To augment the sample size and thus enhance the robustness of our analysis, we incorporated findings from Chinese comparative studies. However, this strategy may potentially increase the risk of language bias. The potential bias will diminish as the literature continues to be enriched with additional randomized controlled trials and comparative studies.

## Conclusion

Given the absence of low risk biased Randomized Controlled Trials (RCTs) up until now, considerable caution is required in interpreting the outcomes due to significant heterogeneity in surgical procedures and reporting of postoperative complications. At present, the Hybrid Hernia Repair (HHR) technique does not appear to offer a distinct advantage over the Laparoscopic Hernia Repair (LHR) method in terms of mitigating surgical complications, except for a lower postoperative seroma incidence. Surgeons with significant expertise may avoid incidental conversions or intentional hybrid procedures. Future research should aim to conduct low-risk biased RCTs to clarify these findings and establish the optimal surgical approach for Incisional Ventral Hernias (IVH).

### Supplementary Information

**Additional file 1: Supplementary Table S1.** The Risk of bias domains (ROBINS-I) of included studies. **Supplementary Table S2.** The Cochrane risk of bias tool for assessing risk of bias in included studies. **Supplementary Table S3.** Source of the Chinese studies included this analysis

## Data Availability

All data generated or analyzed during this study are included in this published article.
