# Supplementary material for: Comparative effectiveness of hybrid and laparoscopic techniques for repairing complex incisional ventral hernias: a systematic review and meta-analysis
Source: BMC Surg. 2023 Nov 16;23:346. doi: 10.1186/s12893-023-02254-6 (PMC10652588; doi:10.1186/s12893-023-02254-6)
Supplement: Supplementary file 1 — Additional file 1: Supplementary Table S1. The Risk of bias domains (ROBINS-I) of included studies. Supplementary Table S2. The Cochrane risk of bias tool for assessing risk of bias in included studies. Supplementary Table S3. Source of the Chinese studies included this analysis [file 12893_2023_2254_MOESM1_ESM.docx]

**Supplementary Information**

The following is the supplementary data to this article:

**Supplementary file 1: Table S1. The Risk of bias domains (ROBINS-I) of included studies**

| Study | Bias Domain | | | | | | | Judgement  (Overall risk of bias) |
| --- | --- | --- | --- | --- | --- | --- | --- | --- |
|  | Bias due to confounding | Bias in selection of participants into study | Bias in classification of interventions | Bias due to deviations from intended intervention | Bias due to missing data | Bias in measurement of outcomes | Bias in selection of the reported result |  |
| Deng X, et al.,2013 | Moderate | Low | Low | Low | Low | Low | Low | Moderate |
| Taqi M, et al.,2013 | Moderate | Moderate | Low | Moderate | Moderate | Moderate | Moderate | Moderate |
| Zhu et al.,2014] | Moderate | Low | Low | Low | Low | Low | Moderate | Moderate |
| Ozturk G, et al.,2015 | Moderate | Low | Low | Low | Low | Low | Low | Moderate |
| Ye J, et al.,2015 | Low | Low | Low | Low | Moderate | Low | Low | Moderate |
| Wang J, et al.,2017 | Moderate | Low | Low | Low | Low | Low | Moderate | Moderate |
| Chen G, et al.,2017 | Moderate | Moderate | Low | Moderate | Moderate | Moderate | Moderate | Moderate |
| Ahonen M, et al.,2017 | Low | Moderate | Low | Low | Low | Low | Low | Moderate |
| Halka JT, et al.,2018 | Low | Low | Low | Low | Low | Low | Low | Low |
| Liu Z, et al.,2019 | Moderate | Moderate | Low | Moderate | Moderate | Low | Moderate | Moderate |
| Zhao C, et al.,2019 | Moderate | Low | Low | Moderate | NI | Moderate | Moderate | Moderate |
| Tian G, et al.,2020 | Moderate | Low | Low | Moderate | Moderate | Low | Low | Moderate |
| Yang S, et al.,2022 | Low | Low | Low | Moderate | Low | Low | Low | Moderate |

Low, low risk of bias; Moderate, moderate risk of bias; NI, no information.

**Supplementary file 2: Table S2. The Cochrane risk of bias tool for assessing risk of bias in included studies**

| Study | Bias domain | | | | | | | Judgement  （Overall risk of bias） |
| --- | --- | --- | --- | --- | --- | --- | --- | --- |
|  | Selection bias | | Performance bias | Detection bias | Attrition bias | Reporting bias | Other bias |  |
|  | Random sequence generation | Allocation concealment | Blinding of participants and personnel | Blinding of outcome assessment | Incomplete outcome data | Selective reporting | Anything else, ideally prespecified |  |
| Ahonen M, et al.,2018,2020 | Low | Low | Low | Unclear | Low | Low | Low | Low |

Low, Low risk of bias; Unclear, Unclear risk of bias.

**Supplementary file 1: Table S3. Source of the Chinese studies included this analysis**

| Studies | Chinese Title | DOI | URL |
| --- | --- | --- | --- |
| Deng X, et al.,2013 | 切口疝的“杂交”修复技术(附40例报告) | 10.3969/j.issn.1007-9610.2013.03.010 | https://kns.cnki.net/kcms/detail/detail.aspx?FileName=WKLL201303016&DbName=CJFQ2013 |
| Taqi M, et al.,2013 | 腹腔镜腹壁切口疝修补术25例报告 | 10.13499/j.cnki.fqjwkzz.2013.04.014 | https://dzj-prod-1.oss-cn-shanghai.aliyuncs.com/2017/09/26/977008537fed4d79b421fa2d01bbf8b9.pdf |
| Zhu et al.,2014] | 杂交技术治疗巨大腹壁切口疝临床疗效分析 | 10.7504/CJPS.ISSN1005-2208.2014.05.18 | http://www.wanfangdata.com.cn/details/detail.do?_type=perio&id=zgsywkzz201405025 |
| Ye J, et al.,2015 | 杂交修补技术与腹腔镜腹腔内补片植入术治疗腹壁切口疝的疗效比较 | 10.13499/j.cnki.fqjwkzz.2015.11.866 | https://kns.cnki.net/kcms/detail/detail.aspx?FileName=FQJW201511024&DbName=CJFQ2015 |
| Wang J, et al.,2017 | 腹腔镜或杂交技术修补大型、巨大型腹壁切口疝的技巧 | 10.3969/j.issn.1009-6604.2017.03.024 | https://d.wanfangdata.com.cn/periodical/ChlQZXJpb2RpY2FsQ0hJTmV3UzIwMjEwNDE1EhV6aG9uZ2d3Y3drenoyMDE3MDMwMjQaCGs2dW5zNzNq |
| Chen G, et al.,2017 | 腹腔镜与杂交技术治疗腹壁切口疝70例分析 | 10.3760/cma.j.issn.1007-631X.2017.12.004 | https://kns.cnki.net/kcms/detail/detail.aspx?FileName=ZHPZ201712005&DbName=ZHYX2017 |
| Liu Z, et al.,2019 | 腹壁切口疝79例患者的诊治体会 | N/A | https://kns.cnki.net/kcms/detail/detail.aspx?FileName=LNYX201903009&DbName=CJFQ2019 |
| Zhao C, et al.,2019 | 杂交手术与腹腔镜下修补术治疗腹壁切口疝的临床研究 | 10.3877/cma.j.issn.1674-392X.2019.06.014 | https://d.wanfangdata.com.cn/periodical/ChlQZXJpb2RpY2FsQ0hJTmV3UzIwMjMwODMxEhN6aHNoZmJ3a3p6MjAxOTA2MDE0Ggh5eHJnMmhnMQ%3D%3D |
| Tian G, et al.,2020 | 开放手术辅助腹腔镜与完全腹腔镜下修补术治疗腹壁切口疝的临床研究 | 10.3877/cma.j.issn.1674-392X.2020.05.004 | https://kns.cnki.net/kcms/detail/detail.aspx?FileName=ZSFD202005004&DbName=CJFQ2020 |

N/A: Not available.
